# CMR feature tracking strain patterns and their association with circulating cardiac biomarkers in patients with hypertrophic cardiomyopathy

**DOI:** 10.1007/s00392-021-01848-5

**Published:** 2021-03-29

**Authors:** Ersin Cavus, Kai Muellerleile, Samuel Schellert, Jan Schneider, Enver Tahir, Celeste Chevalier, Charlotte Jahnke, Ulf K. Radunski, Gerhard Adam, Paulus Kirchhof, Stefan Blankenberg, Gunnar K. Lund, Maxim Avanesov, Monica Patten

**Affiliations:** 1 Clinic of Cardiology, University Heart and Vascular Center Hamburg Eppendorf, Martinistr.52, 20246 Hamburg, Germany; 2 Department of Diagnostic and Interventional Radiology, University Hospital Hamburg Eppendorf, Hamburg, Germany; 3 Department of Cardiology and Angiology, Regio Clinics, Elmshorn and Pinneberg, Germany; 4 Department of Radiology, AK Segeberger Kliniken GmbH, Bad Segeberg, Germany

**Keywords:** Strain imaging, CMR feature tracking strain, Hypertrophic cardiomyopathy, Cardiac biomarker

## Abstract

**Aims:**

CMR feature tracking strain (CMR-FT) provides prognostic information. However, there is a paucity of data in hypertrophic cardiomyopathy (HCM). We sought to analyze global CMR-FT parameters in all four cardiac chambers and to assess associations with NT-proBNP and cardiac troponin T (hsTnT) in patients with HCM.

**Methods:**

This retrospective study included 144 HCM patients and 16 healthy controls with CMR at 1.5 T. Analyses were performed on standard steady-state free precession cine (SSFP) CMR data using a commercially available software. Global left ventricular (LV) strain was assessed as longitudinal (LV_LAX-_GLS), circumferential (LV_LAX-_GCS) and radial strain (LV_LAX-_GRS) on long -axis (LAX) and as LV_SAX_-GCS and LV_SAX_-GRS on short- axis (SAX). Right ventricular (RV-GLS), left atrial (LA-GLS) and right atrial (RA-GLS) strain were assessed on LAX.

**Results:**

We found LV_LAX_-GLS [− 18.9 (− 22.0, − 16.0), − 23.5 (− 25.5, − 22.0) %, *p* = 0.0001), LV_SAX_-GRS [86.8 (65.9–115.5), 119.6 (91.3–143.7) %, *p* = 0.001] and LA_LAX_-GLS [LA_2CH_-GLS 29.2 (19.1–37.7), LA_2CH_-GLS 38.2 (34.3–47.1) %, *p* = 0.0036; LA_4CH_-GLS 22.4 (14.6–30.7) vs. LA_4CH_-GLS 33.4 (28.4–37.3) %, *p* = 0.0033] to be impaired in HCM compared to healthy controls despite normal LVEF. Furthermore, LV and LA strain parameters were impaired in HCM with elevated NT-proBNP and/or hsTnT, despite preserved LVEF compared to HCM with normal biomarker levels. There was a moderate correlation of LV and LA CMR-FT with levels of NT-proBNP and hsTnT.

**Conclusion:**

CMR-FT reveals LV and LA dysfunction in HCM despite normal LVEF. The association between impaired LV strain and elevated NT-proBNP and hsTnT indicates a link between unapparent functional abnormalities and disease severity in HCM.

**Graphic abstract:**

Typical CMR-FT findings in patients with hypertrophic cardiomyopathy
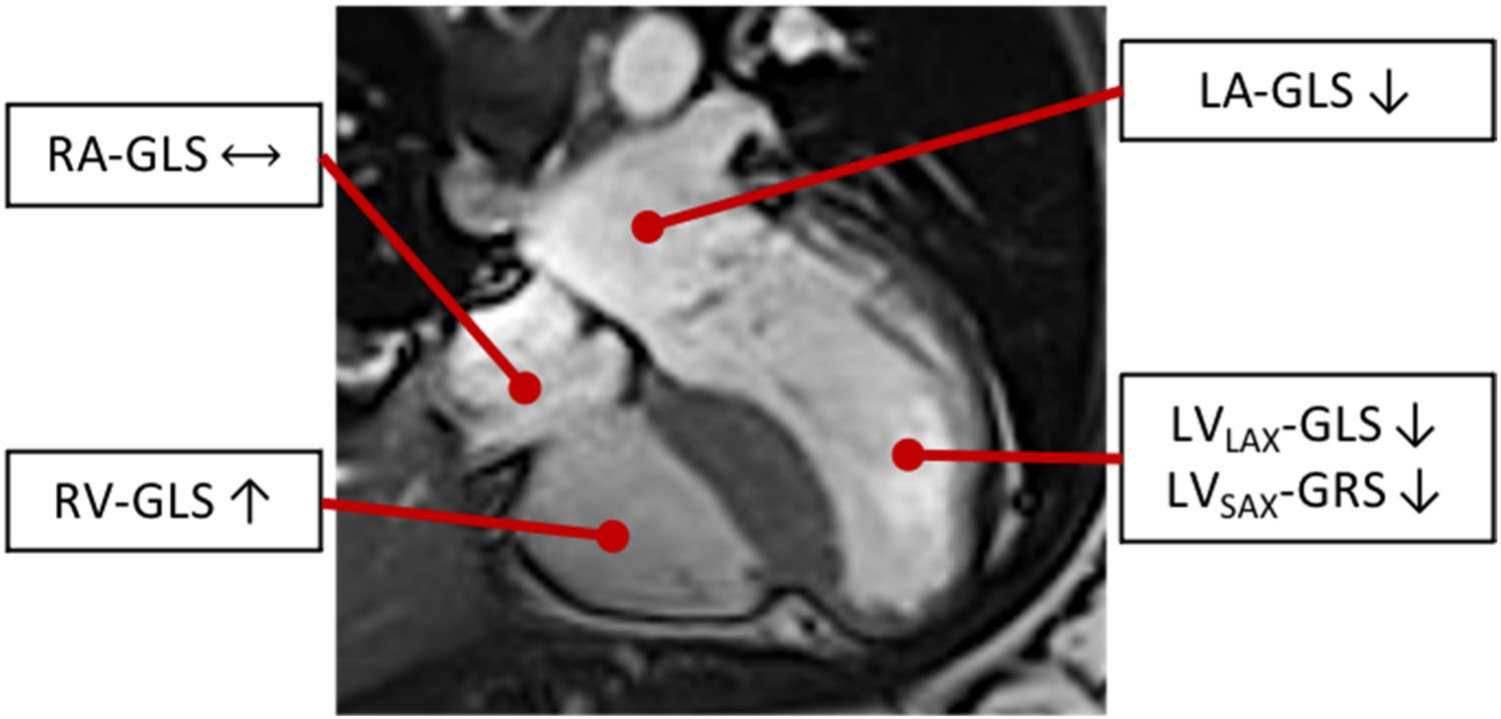

## Background

Hypertrophic cardiomyopathy (HCM) is characterized by inappropriate and mostly asymmetric left ventricular (LV) hypertrophy, resulting from disarrays of fibers and fascicles, dysmorphic myocytes and accumulation of myocardial fibrosis [1]. It represents the most frequent cause of sudden cardiac death in young people, thus early diagnosis and initiation of therapy can prevent life-threatening events [2]. Nevertheless, assessing myocardial function in HCM by cardiac imaging is challenging since conventional parameters, i.e. left ventricular ejection fraction (LVEF), are often preserved or supernormal in HCM patients despite clinical heart failure symptoms [2]. In the past, echocardiography-based strain analyses (speckle tracking imaging (STI)) were able to detect regional and global myocardial dysfunction in patients with HCM with a superior prognostic value for major adverse cardiac events compared to LVEF assessment [3]. In particular, impaired global longitudinal strain (LV_LAX_GLS), was associated with major cardiovascular events in HCM [4]. CMR feature tracking strain (CMR-FT) has the ability to assess early changes in myocardial mechanics more sensitive compared to echocardiography due to the excellent image quality across the complete left and right ventricle of steady-state free precession (SSFP) cine CMR [5]. CMR-FT has been used to assess LV strain in several studies in recent years, indicating incremental prognostic information in different cardiac diseases such as dilatative cardiomyopathy, ischemic cardiomyopathy, and HCM [6]. However, most available study populations were small and a comprehensive approach in HCM including all cardiac chambers is missing. The aim of our retrospective study was to analyze global CMR-FT strain parameters in all four cardiac chambers of HCM patients. Furthermore, we aimed to assess potential associations with well-established cardiac biomarkers such as NT-proBNP and troponin T (hsTnT).

## Methods

### Patients and controls

This retrospective study included 144 consecutive patients with HCM and 16 healthy subjects as controls. HCM patients were outpatients, in stable health condition and underwent clinically indicated CMR between Jun 2006 and Nov 2017. HCM was defined by a wall thickness ≥ 15 mm in one or more LV myocardial segments according to current guidelines of the ESC [2]. Exclusion criteria included coronary artery disease, relevant valvular dysfunction, post-operative status (myectomy, alcohol septal ablation), glomerular filtration rate (GFR) < 30 ml/min/1.73 m^2^. The control group consisted of recently published healthy individuals [7]. Diastolic dysfunction (DD) was assessed and categorized by echocardiography in agreement with current guidelines [8]. Blood samples of all participants were obtained routinely ± 3 days before/after CMR. Troponin T was measured by a high-sensitive assay and an established cutoff was applied with ≥ 14 pg/mL using the 99th percentile for defining abnormal values [9]. The NT-proBNP cutoff was set at 125 pg/mL according to the current guidelines [10]. The study followed the principles outlined in the Declaration of Helsinki and was approved by the local ethics committee. All patients and controls gave their written informed consent to use CMR information for research purposes.

### CMR protocol

Clinically indicated CMR was performed on a 1.5-T scanner (Achieva, Philips Medical Systems, Best, the Netherlands). For the assessment of LV volumes and function, standard retrospectively gated SSFP cine CMR was performed in short- and in long-axis [two-chamber (2CH), three-chamber (3CH), four-chamber view (4CH)]. Typical imaging parameters were: voxel size 1.36 × 1.36 × 6 mm^3^, echo time = 1.67 ms, time to repetition = 3.34 ms, flip angle = 60°, parallel acquisition technique = SENSE. Late gadolinium enhancement (LGE) images were acquired using a phase-sensitive inversion recovery (PSIR) sequence at least ten minutes after bolus injection of contrast media, 0.075 mmol/kg Gd-BOPTA (MultiHance^®^). Imaging parameters were as follows: voxel size 1.36 × 1.36 × 8 mm^3^, echo time = 2.40 ms, time to repetition = 5.50 ms, flip angle = 15°. Since T1 mapping was routinely introduced not until 2011 in our institution, we did not include T1 mapping in this analysis.

### CMR data analysis

CMR data analysis was performed by two trained observers who were blinded to all clinical information. Commercially available post-processing software (Medis Suite MR, QMass ver. 8.1.74.2, QStrain ver. 2.0.70.2, Leiden, The Netherlands) was used to assess volumes and function of all cardiac chambers. LV and RV volumes, as well as LV mass and LV maximal wall thickness, were obtained from cine SAX. LA and RA volumetry were obtained from cine LAX as recommended [11].

CMR-FT measurements were performed using the QStrain application of Medis Suite MR [12]. To generate the most accurate tracing points, endo- and epicardial contours were manually traced in QMass and then copied to QStrain. In agreement with current recommendations LV strain was based on endo- and epicardial contours in short- and long axis, whereas RV, LA and RA contours were tracked from endocardium in LAX [5], [13–16]. Global CMR-FT was assessed as longitudinal (GLS), radial (GRS) and circumferential strain (GCS) in LV; in LA, RA and RV GLS were assessed. Negative values represent shortening of myocardium and positive values represent thickening referred to the direction of the deformation process (longitudinal, radial, circumferential). LA/RA maximum was defined at end-systole and LA/RA minimum at end-diastole. LGE was assessed semi-quantitatively as recommended for clinical indications [11] on PSIR images according to the 17-segment model of the AHA [17].

### Statistical analysis

Statistical analysis was performed using MedCalc for Windows, version 12.7.7.0 (MedCalc Software, Ostend, Belgium). Continuous data are presented as median and IQR. Categorical data are presented as numbers and percentage. Inter-observer agreement was assessed by intra-class correlation coefficients (ICC) and ICC values indicated poor (< 0.5), moderate (0.5–0.75), good (0.75–0.9) or excellent (> 0.9) reliability [18]. Continuous data were compared using the Mann–Whitney test. Categorical data were analyzed using the chi-quadrat test or Fisher exact test, where appropriate. Correlations between continuous parameters were analyzed by spearman’s correlation coefficient (rho). Statistical significance was set to *p* < 0.05 without correction for multiple testing.

## Results

### Inter-observer agreement CMR-FT strain

Inter-observer agreement of CMR-FT strain parameters was as follows: ICC LV_LAX_-GLS 0.95 (0.93, 0.96), LV_LAX_-GCS 0.84 (0.78, 0.89), LV_LAX_-GRS 0.40 (0.17, 0.57), LV_SAX_-GRS 0.89 (0.85, 0.92), LV_SAX_-GCS 0.66 (0.53, 0.76), LA_2CH_-GLS 0.97 (0.96, 0.98), LA_4CH_-GLS 0.97 (0.95, 0.98), RV-GLS 0.94 (0.92, 0.96), and RA-GLS 0.94 (0.91, 0.95).

### HCM patients and controls

**Fig. 1:**
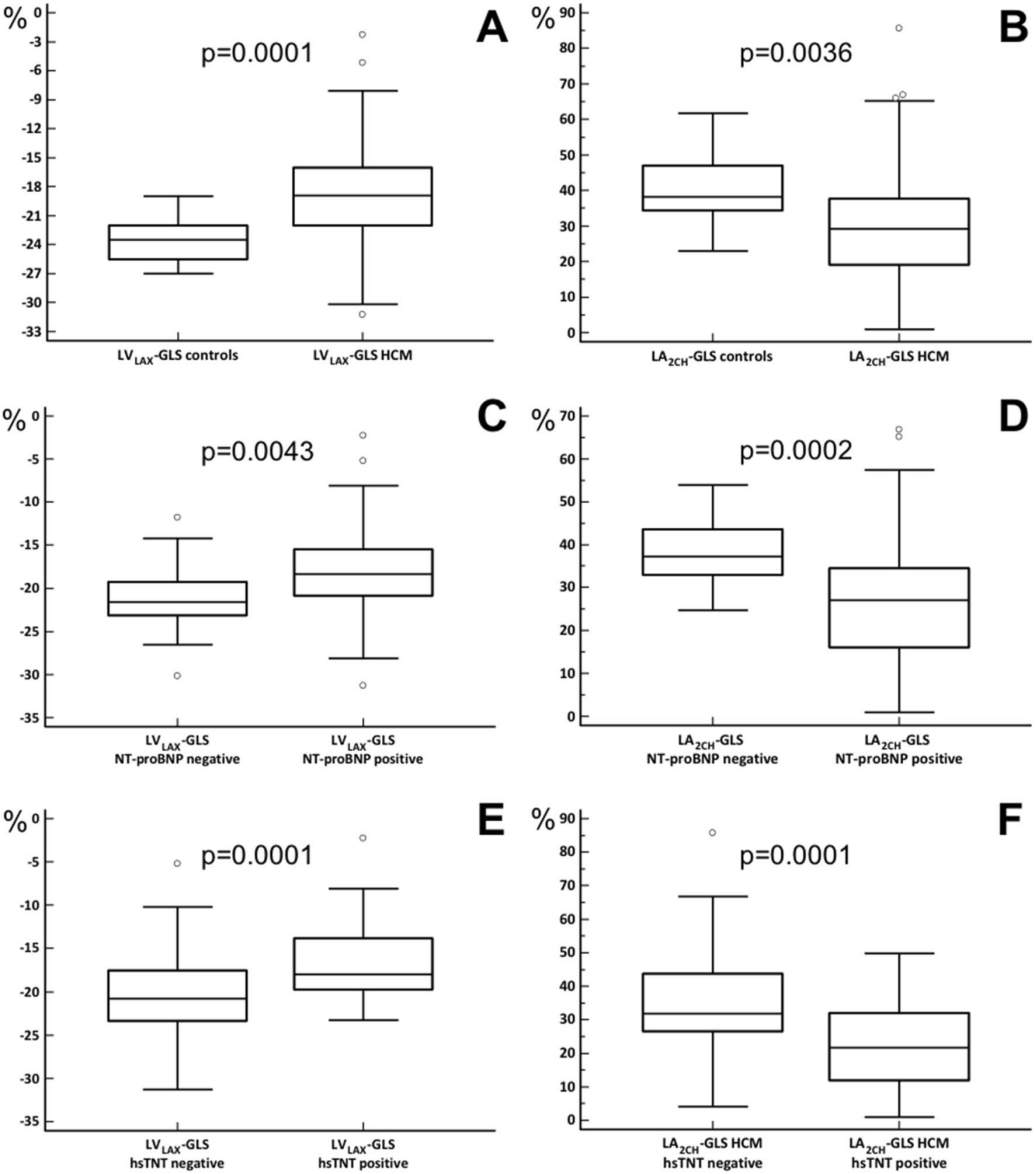
Distribution of LV and LA strain. Box–Whisker plots of median LV_LAX_-GLS and LA_2CH_-GLS in controls and HCM (**a**, **b**) and in HCM with negative (< 125 pg/mL) NT-proBNP blood levels compared to HCM with elevated (positive) NT-proBNP levels (**c**, **d**) as well as in HCM with negative hsTnT (< 14 pg/mL) or elevated (positive) hsTnT (**e**, **f**)

LV Mass index (LVMi), maximal LV wall thickness (LVWT) and median LA volumes were significantly higher in HCM patients compared to controls (Table 1). Eighty-seven (91.6%) of 95 HCM patients with sufficient echocardiography had diastolic dysfunction ≥ II (Table 2). Median LV_LAX_-GLS of HCM patients was significantly lower compared to controls [− 18.9 (− 22.0, − 16.0), − 23.5 (− 25.5, − 22.0) %, *p* = 0.0001; Table 1, Fig. 1). Furthermore, median LV_SAX_-GRS, LA_2CH_-GLS and LA_4CH_-GLS were significantly lower in the HCM group compared to controls (Table 1, Fig. 1). In contrast, median RV-GLS was significantly higher in HCM compared to controls [− 38.0 (− 43.6, − 33.9), − 31.4 (− 34.3, − 28.3) %, *p* = 0.0002; Table 1).

**Table 1:** Clinical characteristics

Parameter, unit	Controls (*n* = 16)	HCM (*n* = 144)	*p* value
Age, years	51 (46–58)	55 (43–64)	0.2191
Male, *n*, (%)	8 (50)	88 (61)	0.4277
Heart rate, bpm	61 (57–76)	65 (59–73)	0.6327
Height, m	1.73 (1.63–1.76)	1.74 (1.66–1.82)	0.5636
Weight, kg	78 (70–88)	82 (71–90)	0.4110
Creatinine, mg/dL	0.79 (0.66–0.90)	0.9 (0.80–1.10)	**0.0220**
GFR, mL/min	97 (91–109)	78 (70–88)	**0.0037**
Creatine kinase, U/L	127 (65–184)	115 (79.5–171)	0.8737
hsTroponin T, pg/mL	3 (3–4)	12 (7–21.5)^a^	**< 0.0001**
NT-proBNP, pg/mL	46 (32–97)	581 (227–1542)^b^	**< 0.0001**
Volumes and functions			
LVEF, %	65 (59–68)	64 (58–71)	1.0000
LVEDVi, mL/m^2^	85 (79–90)	88 (78–99)	0.6842
LVESVi, mL/m^2^	29 (26–38)	30 (24–40)	0.9410
LV mass index, g/m^2^	45 (36–58)	76 (57–95)	**< 0.0001**
Maximal LVWT, mm	8.9 (8.2–9.9)	17.9 (15.6–21.4)	**< 0.0001**
RVEF, %	62 (57–65)	64 (58–68)	0.5153
RVEDVi, mL/m^2^	80 (67–89)	73 (64–88)	0.4712
RVESVi, mL/m^2^	29 (23–35)	27 (21–35)	0.5832
LAEDVi, mL/m^2^	14 (12–17)	36 (22–56)	**< 0.0001**
LAESVi, mL/m^2^	39 (31–41)	54 (41–73)	**0.0002**
RAEDVi, mL/m^2^	22 (16–25)	22 (16–32)	0.4050
RAESVi, mL/m^2^	40 (28–43)	38 (27–48)	1.0000
LGE presence, *n*, (%)	0/16 (0)	104/143 (73)	**< 0.0001**
LGE extent, *n* (amount of LGE segments in %)	0/272 (0)	308/2448 (12.6)	**< 0.0001**
Atrial fibrillation, *n*, (%)	0	7(4.9)	**< 0.0001**
HCM-SCD-score, %	0	3.3 (2.2–5.5)	**< 0.0001**
Strain parameters, %			
LV_LAX_-GLS	− 23.5 (− 25.5, − 22.0)	− 18.9 (− 22.0, − 16.0)	**0.0001**
LV_LAX_-GCS	− 25.0 (− 26.9, − 23.2)	− 22.9 (− 26.4, − 20.0)	0.0848
LV_LAX_-GRS	96.4 (75.4–114.1)	87.5 (68.9–107.4)	0.3113
LV_SAX_-GCS	− 23.3 (− 27.9, − 21.1)	22.1 (− 24.8, -18.3)	0.1696
LV_SAX_-GRS	119.6 (91.3–143.7)	86.8 (65.9–115.5)	**0.0014**
LA_2CH_-GLS	38.2 (34.3–47.1)	29.2 (19.1–37.7)	**0.0036**
LA_4CH_-GLS	33.4 (28.4–37.3)	22.4 (14.6–30.7)	**0.0033**
RV-GLS	− 31.4 (− 34.3, − 28.3)	-38.0 (− 43.6, − 33.9)	**0.0002**
RA-GLS	29.8 (24.1–35.1)	31.1 (23.5–39.2)	0.6959

Statistical significance was defined as *p* < 0.05. Significant results are highlighted in bold

Values are median [first (Q1) and third (Q3) quartiles] for continuous and *n* (% of total column number) for categorical data. *HCM* indicates hypertrophic cardiomyopathy, *bpm*, beats per minute, *hs* high-sensitive, *NT* *N*-terminal, *LV* left ventricular, *RV* right ventricular, *LA* left atrial, *RA* right atrial, *EF* ejection fraction, *EDVi* end-diastolic volume index, *ESVi* end-systolic volume index, *LVWT* left ventricular wall thickness, *LGE* late gadolinium enhancement, *SCD-Score* sudden cardiac death score, *LAX* longitudinal axis, *SAX* short axis, *GLS* global longitudinal strain, *GCS* global circumferential strain, *GRS* global radial strain, *2CH* two-chamber view, *4CH* four-chamber view

^a^hsTnT was available in *n* = 119

^b^NT-proBNP was available in *n* = 124

**Table 2:** Clinical and CMR characteristics of HCM patients with and without elevated NT-proBNP

Parameter, unit	NT-proBNP < 125 pg/mL (*n* = 20)	NT-proBNP ≥ 125 pg/mL (*n* = 104)	*p* value
Age, years	49 (38–57)	57 (45–65)	0.0631
Male, *n*, (%)	18 (90)	60 (57.7)	**0.0054**
Heart rate, bpm	65 (57–72)	66 (59–73)	0.4713
Height, m	1.79 (1.72–1.84)	1.73 (1.65–1.82)	0.0616
Weight, kg	88 (81–92)	82 (73–90)	0.1065
Creatinine, mg/dL	1.0 (0.9–1.2)	0.9 (0.8–1.1)	**0.0268**
GFR, mL/min	79 (66–97)	80 (68–100)	0.5543
Creatine kinase, U/L	138 (118–170)	108 (73–169)	0.1108
hsTroponin T, pg/mL	5 (3–8)	13 (8–27)	** < 0.0001**
Volumes and functions			
LVEF, %	67 (61–72)	64 (57–69)	0.0569
LVEDVi, mL/m^2^	86 (77–104)	89 (79–99)	0.9729
LVESVi, mL/m^2^	28 (21–39)	33 (27–42)	0.1237
LV mass index, g/m^2^	71 (57–86)	79 (60–98)	0.0796
Maximal LVWT, mm	16.3 (14.7–17.7)	18.6 (15.8–21.4)	0.0182
RVEF, %	63 (58–67)	64 (57–68)	0.9864
RVEDVi, mL/m^2^	85 (68–102)	73 (63–88)	0.0267
RVESVi, mL/m^2^	32 (26–39)	27 (22–36)	0.1006
LAEDVi, mL/m^2^	23 (17–26)	38 (26–58)	**0.0001**
LAESVi, mL/m^2^	49 (42–59)	57 (43–79)	0.1747
RAEDVi, mL/m^2^	23 (17–27)	22 (16–34)	0.6828
RAESVi, mL/m^2^	42 (36–48)	36 (27–49)	0.1707
Normal diastolic Function, *n*, (%)	0/12 (0)	1/83 (1.2)	1.0000
DD I°, *n*, (%)	0/12 (0)	7/83 (8.4)	0.5901
DD II°, *n*, (%)	11/12(92)	61/83 (73.5)	0.2821
DD III°, *n*, (%)	1/12 (8)	14/83	0.6837
DD ≥ II°, *n*, (%)	12/12 (100)	75/83 (90.3)	0.5901
NYHA I, *n*, (%)	7/20 (35)	32/99 (32.3)	0.7996
NYHA II, *n*, (%)	11/20 (55)	45/99 (45.5)	0.4703
NYHA III, *n*, (%)	2/20 (10)	22/99 (22.2)	0.3587
NYHA IV, *n*, (%)	0/20 (0)	0/99 (0)	1.0000
LGE presence, *n*, (%)	11/20 (55)	78/104 (75)	0.1013
LGE extent, *n* (amount of LGE segments in %)	19/340 (5.6)	233/1768 (13.2)	** < 0.0001**
Atrial fibrillation, *n*, (%)	0 (0)	4 (3.8)	1.0000
HCM-SCD-Score, %	2.6 (1.8–6.1)	3.5 (2.2–5.0)	0.3939
LVOT obstruction, *n*, (%)	4 (20)	39 (37.5)	0.1990
Strain parameters, %			
LV_LAX_-GLS	− 21.6 (− 23.2, − 19.3)	− 18.4 (− 20.8, − 15.5)	**0.0043**
LV_LAX_-GCS	− 26.1 (− 27.7, − 22.9)	− 22.2 (− 25.5, − 19.2)	**0.0039**
LV_LAX_-GRS	107.0 (82.5–126.1)	84.5 (68.4–104.0)	**0.0198**
LV_SAX_-GCS	− 23.4 (− 25.2, − 21.8)	− 21.3 (− 24.0, − 17.4)	**0.0125**
LV_SAX_-GRS	102.3 (78.0–126.9)	82.0 (62.0–108.5)	**0.0292**
LA_2CH_-GLS	37.2 (33.0–43.7)	27.0 (16.1–34.5)	**0.0002**
LA_4CH_-GLS	28.8 (22.8–41.8)	21.8 (13.2–29.0)	**0.0051**
RV-GLS	− 37.6 (− 44.8, − 32.6)	− 38.0 (− 43.6, − 33.9)	0.6577
RA-GLS	33.7 (28.9–42.0)	30.9 (22.5–39.1)	0.0739

Statistical significance was defined as *p* < 0.05. Significant results are highlighted in bold

Values are median [first (Q1) and third (Q3) quartiles] for continuous and *n* (% of total column number) for categorical data

*HCM* indicates hypertrophic cardiomyopathy, *bpm* beats per minute, *hs* high-sensitive, *NT* *N*-terminal, *LV* left ventricular, *RV* right ventricular, *LA* left atrial, *RA* right atrial, *EF* ejection fraction, *EDVi* end-diastolic volume index, *ESVi* end-systolic volume index, *LVWT* left ventricular wall thickness, *DD* diastolic dysfunction, *NYHA* New York Heart Association, *LGE* late gadolinium enhancement, *SCD-Score* sudden cardiac death score, *LAX* longitudinal axis, *SAX* short axis, *GLS* global longitudinal strain, *GCS* global circumferential strain, *GRS* global radial strain, *2CH* two-chamber view, *4CH* four-chamber view

### NT-proBNP

**Fig. 2:**
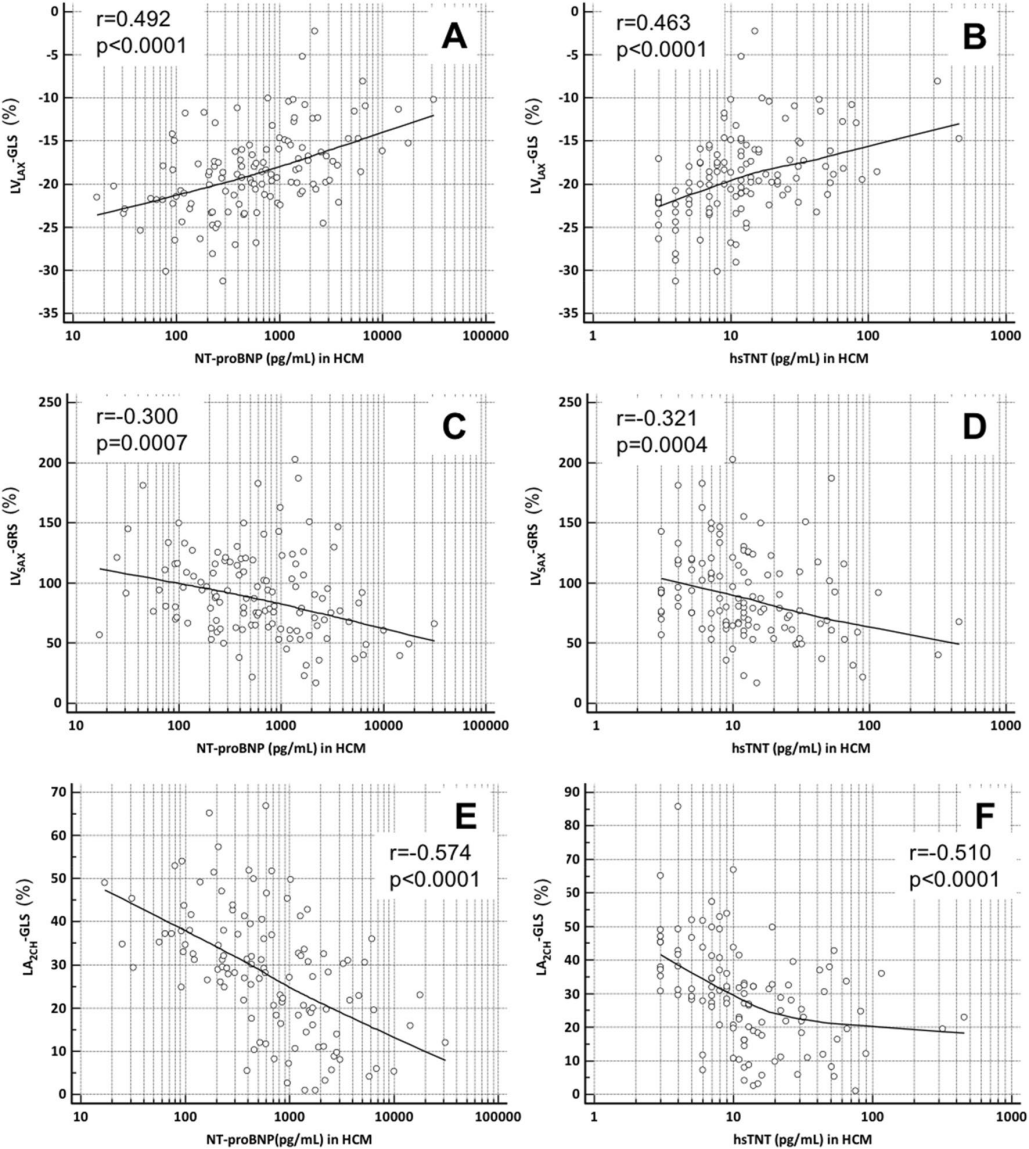
Correlation of LV_LAX_GLS, LV_SAX_GRS and LA_2CH_GLS with NT-proBNP and hsTnT. Scatterplots of LV_LAX_GLS, LV_SAX_GRS, LA_2CH_GLS and circulation biomarkers such as NT-proBNP and high-sensitivity cardiac troponin T (hsTnT). Spearman’s correlations were **a** LV_LAX_GLS and NT-proBNP: *r* = 0.492, *p* < 0.0001; **b** LV_LAX_GLS and hsTnT: *r* = 0.463, *p* < 0.0001; **c** LV_SAX_GRS and NT-proBNP: *r* = − 0.300, *p* = 0.0007; **d** LV_SAX_GRS and hsTnT: *r* = − 0.321, *p* = 0.0004; **e** LA_2CH_GLS and NT-proBNP: *r* = − 0.574, *p* < 0.0001; **f** LA_2CH_GLS and hsTnT: *r* = − 0.510, *p* < 0.0001. Note: logarithmic transformation of *x*-axis

104 (84%) HCM patients had elevated NT-proBNP levels. There were no significant differences in major clinical and conventional CMR characteristics between HCM patients with and without elevated NT-proBNP levels (Table 2). HCM patients with elevated NT-proBNP had a significantly higher extent of LGE. LV_LAX_-GLS, LV_LAX_-GCS, LV_LAX_-GRS, LV_SAX_-GCS, LV_SAX_-GRS and global longitudinal LA strain were all significantly lower in HCM patients with elevated NT-proBNP (Table 2, Fig. 1). There were no significant differences in RA and RV strain between both groups (Table 2). There were significant correlations of NT-proBNP levels with LV_LAX_-GLS and LV_SAX_-GRS (LV_LAX_-GLS: *r* = 0.492, *p* < 0.0001; LV_SAX_-GRS: *r* = − 0.300, *p* = 0.0007, Fig. 2).

### Troponin T

44 HCM patients had elevated hsTnT levels (≥ 14 pg/mL) (Table 3). There were no significant differences in major clinical characteristics, but a significantly lower (but normal) median LVEF as well as higher LV/LA volumes and higher LVMi in HCM patients with elevated hsTnT levels (Table 3). Diastolic dysfunction ≥ stage II was present in the majority of both groups but less frequent in the group with hsTnT ≥ 14 pg/mL (81.3 vs. 96.5%, *p* = 0.0233). The presence of LGE was similar in both groups, but HCM patients with elevated hsTnT had a significantly higher extent of LGE (Table 3). LV_LAX_-GLS, LV_LAX_-GCS, LV_LAX_-GRS, LV_SAX_-GCS, LV_SAX_-GRS and global longitudinal LA strain were all significantly lower in the group of HCM patients with elevated hsTnT (Table 3). LV_LAX_-GLS (*r* = 0.463, *p* < 0.0001) and LV_SAX_-GRS correlated significantly with hsTnT levels (*r* = − 0.321, *p* = 0.0004; Fig. 2).

**Table 3:** Clinical and CMR characteristics of HCM patients with and without elevated hsTnT

Parameter, unit	HCM with hsTnT < 14 pg/mL (*n* = 75)	HCM with hsTnT ≥ 14 pg/mL (*n* = 44)	*p* value
Age, years	53 (41–63)	59 (53–67)	0.0471
Male, *n*, (%)	48/75 (64%)	26/44 (59%)	0.6958
Heart rate, bpm	65 (60–72)	66 (58–76)	0.7557
Height, m	1.74 (1.66–1.82)	1.75 (1.65–1.83)	0.7809
Weight, kg	82 (74–92)	84 (75–92)	0.9079
Creatinine, mg/dL	0.9 (0.75–1.09)	1.0 (0.80–1.16)	0.1255
GFR, mL/min	85 (68–101)	74 (62–99)	0.0375
Creatine kinase, U/L	113 (79–169)	115 (84–174)	0.6362
NT-proBNP, pg/mL	421 (136–961)	1228 (459–2451)	**< 0.0001**
Volumes and functions			
LVEF, %	66 (58–72)	62 (57–66)	**0.0138**
LVEDVi, mL/m^2^	85 (76–96)	92 (81–106)	**0.0445**
LVESVi, mL/m^2^	28 (24–35)	36 (28–46)	**0.0016**
LV Mass Index, g/m^2^	71 (55–86)	83 (70–109)	**0.0013**
Maximal LVWT, mm	17.3 (15.3–20.7)	19.1 (15.9–21.4)	0.1831
RVEF, %	64 (58–68)	64 (53–70)	0.8017
RVEDVi, mL/m^2^	72 (64–87)	74 (65–91)	0.5244
RVESVi, mL/m^2^	26 (23–34)	29 (22–37)	0.4360
LAEDVi, mL/m^2^	27 (20–43)	45 (28–65)	**0.0011**
LAESVi, mL/m^2^	50 (42–63)	64 (48–90)	**0.0115**
RAEDVi, mL/m^2^	20 (16–27)	24 (18–39)	0.0582
RAESVi, mL/m^2^	38 (28–47)	36 (26–58)	0.5691
Normal diastolic function, *n*, (%)	0/57 (0)	1/32 (3.1)	0.3596
DD I°, *n*, (%)	2/57 (3.5)	5/32 (15.6)	0.0930
DD II°, *n*, (%)	46/57 (80.7)	21/32 (65.6)	0.1308
DD III°, *n*, (%)	9/57 (15.8)	5/32 (15.6)	1.0000
Diastol dysfunction ≥ II°, *n*, (%)	55/57 (96.5)	26/32 (81.3)	**0.0233**
NYHA I, *n*, (%)	26/73 (35.6)	13/40 (32.5)	0.8371
NYHA II, *n*, (%)	33/73 (45.2)	19/40 (47.5)	0.8456
NYHA III, *n*, (%)	14/73 (19.2)	8/40 (20)	0.4335
NYHA IV, *n*, (%)	0/73 (0)	0/40 (0)	1.0000
LGE presence, n, (%)	51/74 (69)	35/44 (80)	0.2847
LGE extent, *n* (amount of LGE segments in %)	129/1258 (10)	118/748 (16)	**0.0003**
Atrial fibrillation, *n*, (%)	2/75 (2.7)	5/44 (11.4)	0.1117
HCM-SCD-Score, %	2.9 (2.1–5.1)	3.3 (2.2–5.3)	0.3997
LVOT obstruction, *n*, (%)	26/75(34.7)	15/44 (34.1)	1.0000
Strain parameters, %			
LV_LAX_-GLS	− 20.8 (− 23.4, − 19.0)	− 18.0 (− 19.7, − 13.8)	**0.0001**
LV_LAX_-GCS	− 24.7 (− 26.9, − 22.0)	− 20.5 (− 24.3, − 17.1)	**0.0001**
LV_LAX_-GRS	91.2 (76.4–115.1)	79.8 (58.9–96.4)	**0.0230**
LV_SAX_-GCS	− 22.2 (− 25.5, − 19.7)	− 21.0 (− 23.8, − 16.4)	**0.0217**
LV_SAX_-GRS	93.5 (70.0–119.7)	72.0 (53.3–97.0)	**0.0020**
LA_2CH_-GLS	31.8 (26.5–43.7)	21.8 (11.9–32.0)	**0.0001**
LA_4CH_-GLS	25.3 (18.4–31.6)	16.4(12.6–26.1)	**0.0021**
RV-GLS	− 38.3 (− 44.1, − 33.7)	− 38.3 (− 43.9, − 34.2)	0.9015
RA-GLS	33.9 (26.4–43.1)	26.5 (22.0–32.1)	**0.0011**

Statistical significance was defined as *p* < 0.05. Significant results are highlighted in bold

Values are median [first (Q1) and third (Q3) quartiles] for continuous and *n* (% of total column number) for categorical data

*HCM* indicates hypertrophic cardiomyopathy, *bpm* beats per minute, *hs* high-sensitivity, *NT* *N*-terminal, *LV* left ventricular, *RV* right ventricular, *LA* left atrial, *RA* right atrial, *EF* ejection fraction, *EDVi* end-diastolic volume index, *ESVi* end-systolic volume index, *LVWT* left ventricular wall thickness, *DD* diastolic dysfunction, *NYHA* New York Heart Association, *LGE* late gadolinium enhancement, *SCD-Score* sudden cardiac death score, *LAX* longitudinal axis, *SAX* short axis, *GLS* global longitudinal strain, *GCS* global circumferential strain, *GRS* global radial strain, *2CH* two-chamber view, *4CH* four-chamber view

## Discussion

This study analyzed global myocardial strain patterns in all four cardiac chambers in patients with HCM by CMR-FT strain. The major findings were the following: first, we found LV_LAX_-GLS, LV_SAX_-GRS and LA_LAX_-GLS to be impaired in HCM patients compared to healthy controls despite normal LVEF in both groups (Table 1, Fig. 1). Second, all global LV and LA strain parameters were impaired in HCM patients with elevated NT-proBNP and hsTnT levels, despite preserved LVEF and a similar degree of diastolic dysfunction (Tables 2, 3). Third, we found significant but modest correlations of LV and LA CMR-FT strain with NT-proBNP and hsTnT levels (Fig. 2).

### LV and RV strain in HCM

**Fig. 3:**
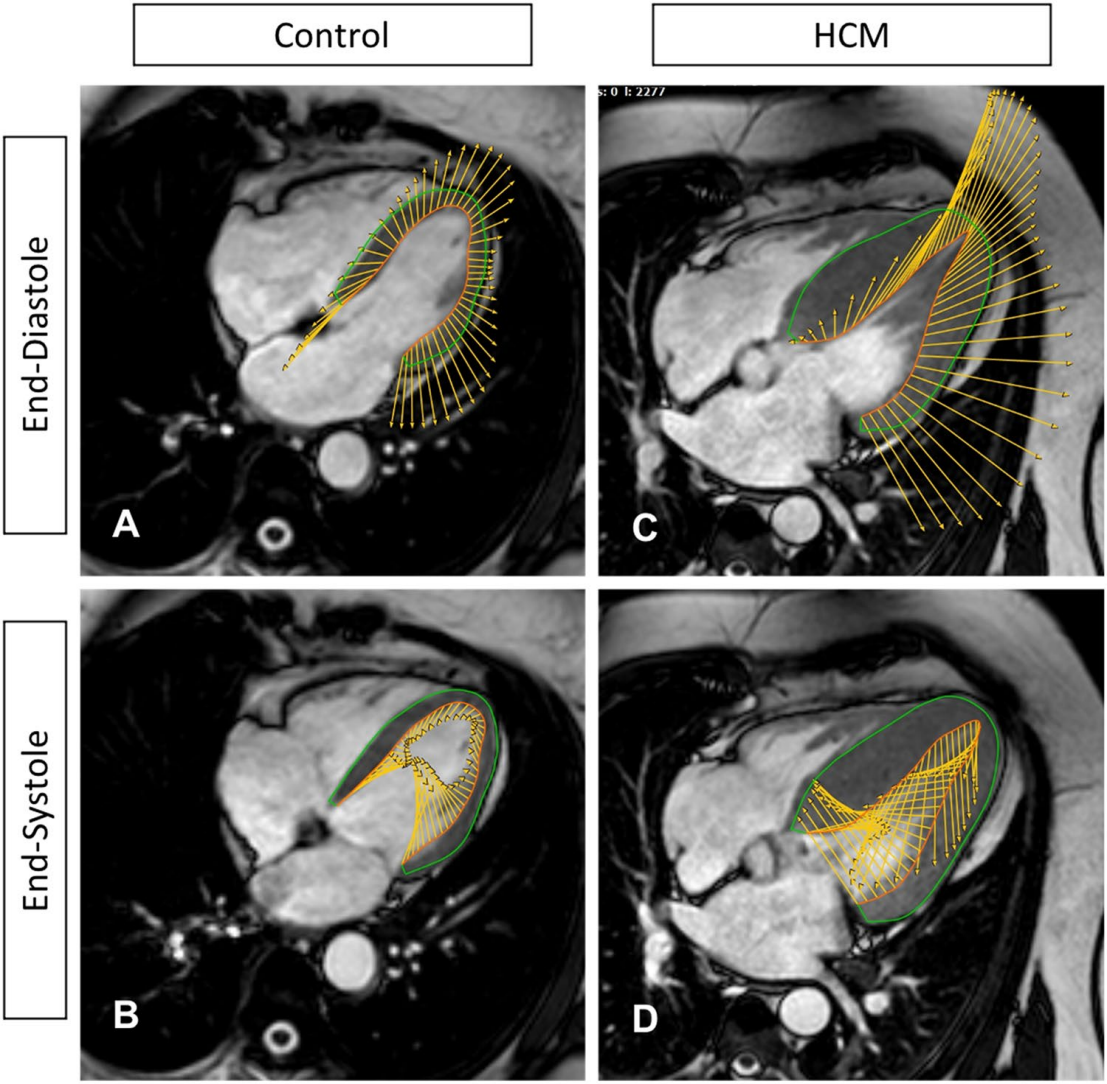
Deformation mechanics in control and in HCM. **a** Diastolic phase in control; LV is relaxing homogeneously. **b** Systolic phase in control; LV is contracting with a shortening in the longitudinal axis. **c** Diastolic phase in HCM; impaired relaxation of the hypertrophied LV septum. **d** Systolic phase in HCM; impaired contraction of the hypertrophied LV septum, compensatory hypercontractile motion of non-hypertrophied LV segments, impaired longitudinal shortening. Note, length of arrows displays relative extent of deformation

**Fig. 4:**
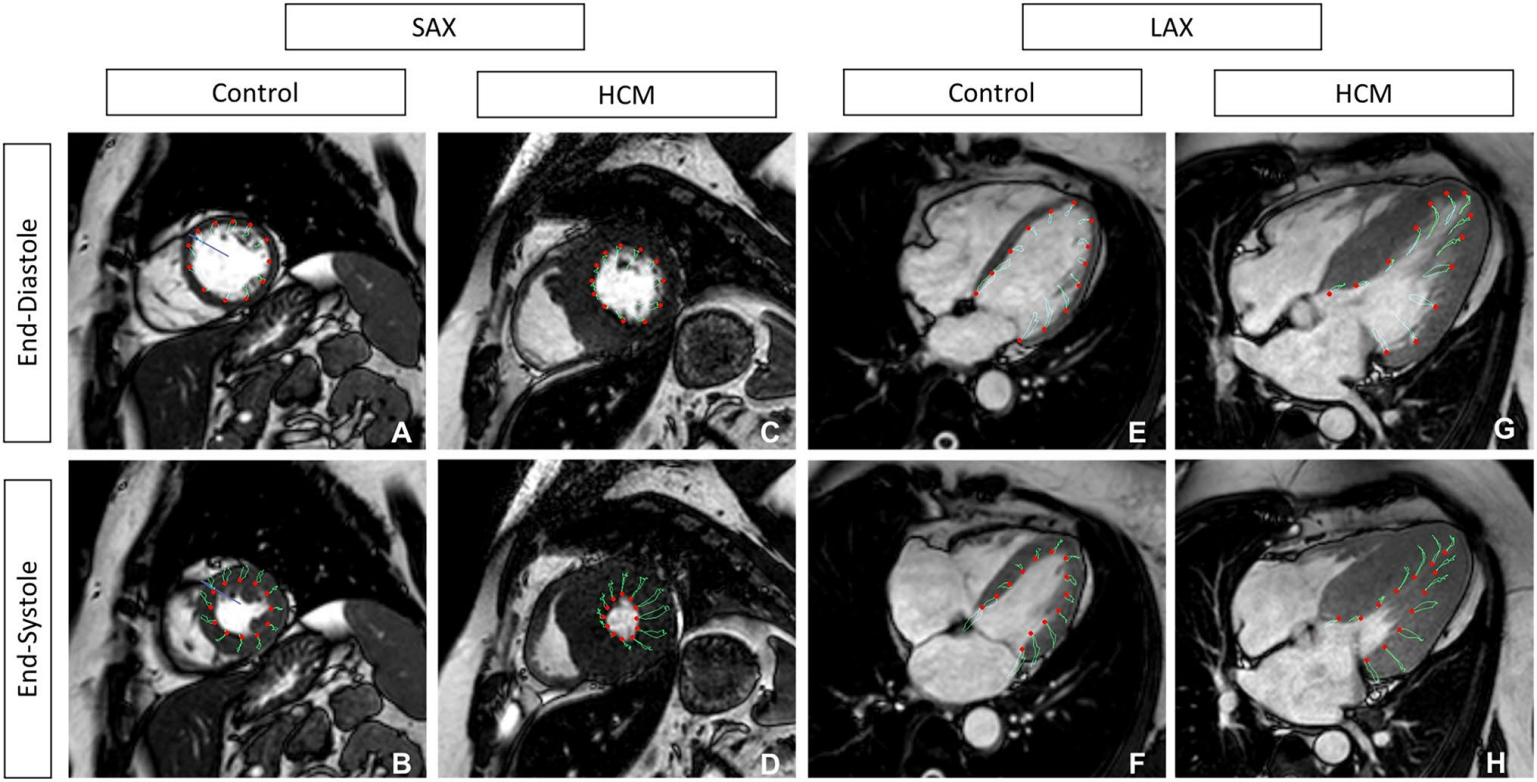
Typical LV-strain findings in HCM in comparison to a control. **a**–**d** Short-axis (SAX) left midventricular (LV) cine images of a control (**a**, **b**) and a HCM patient in end-diastole and end-systole (**c**, **d**). Note, the typical asymmetric septal LV hypertrophy (**c**, **d**). **e**–**h** Long-axis (LAX) LV strain of the same control (**e**, **f**) and the same HCM patient (**g**, **h**). Note, red dots follow green lines and display myocardial deformation within one heart cycle. Septal deformation is lower than i.e., lateral deformation, shown by shorter green lines

We found significantly decreased median LV_LAX_-GLS and LV_SAX_-GRS despite preserved LVEF in HCM patients compared to healthy controls. Although LVEF is by far the most important clinical measure of LV function [19], it tends to overestimate LV systolic function in hypertrophied ventricles [2]. Therefore, more advanced approaches to assess myocardial function in HCM are desirable. CMR-FT offers many different advantages; it traces the cardiac deformation process precisely and reflects local forces (Fig. 3: arrows show direction and proportion of motion), displays disproportional LV thickening (note the reduced amplitude of voxel motion in CMR-FT strain in Fig. 4a–h), is well validated and has been used in a variety of cardiovascular diseases [20]. Moreover, LV_LAX_-GLS and LV_SAX_-GRS provide incremental prognostic information to LVEF in ischemic and non-ischemic cardiac disease [3, 21] and are associated with an increased risk of mortality and poor cardiovascular outcome in HCM [6]. We suspect that the association of strain with myocardial fibrosis [22] and scaring [23] could explain this observation.

In contrast, we found RV-GLS to be higher in HCM compared to controls (Table 1). RV function was neglected in most cardiac diseases for a long time, but in recent years, the awareness is increasing [24, 25]. Hypothetically, the “supra-normal” RV-GLS we found could indicate a compensatory reaction to increased LV filling pressure and requires further investigation. Taken together, our findings suggest that CMR-FT identifies LV and RV functional changes in HCM independent from conventional parameters. CMR-FT contributes to a better understanding of pathophysiology and could potentially improve risk stratification in HCM.

### Atrial strain in HCM

We found that LA_2CH_-GLS and LA_4CH_-GLS were significantly lower in patients with HCM compared to controls. Quantification of LA Strain is a sensitive marker of LV diastolic dysfunction independent of LVEF [26]. In our study, the majority of HCM patients (92%) had advanced diastolic dysfunction (Table 2). Interestingly, Habibi et al. demonstrated that deteriorations in LA-GLS precede the development of HF [27]. It is also known, that LA-GLS is predictive of worsening HF in patients with HCM [28]. Taking these results into account, LA_2CH_-GLS and LA_4CH_-GLS may not only reflect diastolic LV dysfunction in HCM but could also serve as an early predictor of HF and atrial fibrillation risk in HCM patients. Longitudinal studies are necessary in this context. However, we did not find significant difference in RA-GLS between HCM and controls (Table 1). Median RA-GLS was similar to previously described reference values [29]. We assume that the role of RA function in the pathophysiology of HCM is marginal.

### Myocardial strain and the association with cardiac biomarkers

NT-proBNP is a major cardiac biomarker, that is not only used to diagnose HF [10] but also as a powerful prognostic factor in different cardiomyopathies [30]. Cardiac hsTnT is a crucial marker of myocardial injury and is linked to poor outcome in ischemic as well as non-ischemic cardiomyopathies [31, 32]. We found that all global CMR-FT parameters of the LV, but not conventional LVEF and diastolic function, were significantly impaired in HCM patients with elevated biomarker concentrations (Tables 2, 3). Furthermore, we found a significant correlation of biomarker levels with CMR-FT (Fig. 2). These findings are in line with recent STI and CMR-FT studies, in which reduced GLS, GCS and GRS in HCM were independent predictors of poor cardiac outcome, particularly HF [6, 33]. Furthermore, in a recent CMR-FT study Tanacli et al. demonstrated that LV GLS and GCS drop with the severity of HF [34]. Our findings indicate that LV CMR-FT strain reveals occult systolic dysfunction in HCM. Furthermore, diastolic dysfunction is very common in HCM (Table 2) and LA-GLS seems to be a suitable strain parameter to evaluate diastolic dysfunction [26]. In conclusion, alterations in myocardial strain and elevated biomarkers seem to depict patients with more severe disease, independent from conventional assessment such as LVEF, NYHA classification, LVOT obstruction or the SCD risk score. Future studies are necessary to assess the potential prognostic implications of these findings.

### Limitations

This study is a retrospective study with its inherent limitations, such as missing values of cardiac biomarkers in some patients. Furthermore, this study did not include longitudinal, follow-up data and is, therefore, not designed to address a potential incremental value of CMR-FT strain over conventional imaging. Future longitudinal studies are needed to assess a potential incremental prognostic value of CMR-FT over conventional imaging such as the assessment of diastolic dysfunction by echocardiography. To date, CMR-FT strain is often used in addition to conventional imaging parameters since there is currently no clinical application with immediate individual benefit for patients. In particular, there is a substantial overlap in some strain parameters between HCM patients and controls. However, strain reflects the myocardial deformation process much more accurately compared to conventional imaging and may therefore contribute to a better understanding of subclinical pathophysiological myocardial alterations. In addition, T1-mapping/ECV imaging was not available before 2011 in this study population. Therefore, we were not able to systematically address potential associations between strain parameters and quantitative tissue characterization in this study. Long-axis-based GLS is currently perceived as the most robust and reproducible CMR-FT parameter, but the reproducibility of other strain parameters can differ significantly [12, 35]. We found that LV_LAX_-GRS and LV_SAX_-GCS were affected by a poor and moderate inter-observer agreement in our study population, respectively. However, there were good to excellent inter-observer agreements for all other CMR-FT-derived measurements in this study population, which supports the reliability of the major findings of this work.

## Conclusion

CMR-FT reveals LV and LA dysfunction in HCM patients despite normal LVEF. The association between impaired LV strain and elevated NT-proBNP and hsTnT levels indicates a link between unapparent functional abnormalities and disease severity in HCM.

## Abbreviations

CMR-FT: Cardiac magnetic resonance feature tracking strain
HCM: Hypertrophic cardiomyopathy
HF: Heart failure
hsTnT: High-sensitivity troponin T
LA_2CH_-GLS: Left atrium two-chamber global longitudinal strain
LA_4CH_-GLS: Left atrium four-chamber global longitudinal strain
LGE: Late gadolinium enhancement
LVEF: Left ventricular ejection fraction
LV_LAX_GCS: Left ventricular long-axis global circumferential strain
LV_LAX_GLS: Left ventricular long-axis global longitudinal strain
LV_LAX_GRS: Left ventricular long-axis global radial strain
LV_SAX_GCS: Left ventricular short-axis global circumferential strain
LV_SAX_GRS: Left ventricular short-axis global radial strain
NT-proBNP: *N*-terminal prohormone of the brain natriuretic peptide
PSIR: Phase-sensitive inversion recovery
RA-GLS: Right atrium global longitudinal strain
RV-GLS: Right ventricle global longitudinal strain
SCD: Sudden cardiac death
SSFP: Steady-state free precession
STI: Speckle tracking imaging
